# Concurrent training is associated with enhanced post-exercise ceramide clearance and fat oxidation capacity in individuals with type 2 diabetes following lifestyle intervention

**DOI:** 10.1007/s00125-026-06820-4

**Published:** 2026-08-05

**Authors:** Mikael Flockhart, Michael Svensson, Emad Ehtesham, Mats Ryberg, Andreas Stomby, Torbjörn Åkerfeldt, Julia Otten, Tommy Olsson, Elin Chorell

**Affiliations:** 1 Department of Public Health and Clinical Medicine, Umeå University, Umeå, Sweden; 2 Department of Community Medicine and Rehabilitation, Section of Sports Medicine, Umeå University, Umeå, Sweden; 3 Department of Medical Sciences, Clinical Chemistry, Uppsala University, Uppsala, Sweden

**Keywords:** Ceramides, Concurrent training, Exercise training, Lipidomics, Sphingolipids, Triacylglycerols, Type 2 diabetes

## Abstract

**Aim/hypothesis:**

This study aimed to investigate whether concurrent training, when added to a palaeolithic-type diet-induced weight loss intervention, is associated with altered physiological responses and circulating lipid dynamics during exercise and recovery in individuals with type 2 diabetes.

**Methods:**

In a previously reported clinical trial, individuals with overweight or obesity who had type 2 diabetes (*n*=27) were randomised to either 12 weeks of palaeolithic-type diet (PD) or PD and supervised concurrent exercise training three times weekly (PD+E). Physiological and glycaemic responses to exercise and recovery were monitored during an exercise test at baseline and following the intervention. This secondary analysis focuses on a subgroup (*n*=21), in which plasma samples were collected before and after exercise, and after 30 min of recovery, and lipid profiles were analysed using mass spectrometry-based lipidomics.

**Results:**

Glycaemic management during and after the exercise test improved similarly in both groups, whereas only the PD+E group showed improvements in cardiometabolic fitness. In plasma samples obtained at rest before exercise, the intervention reduced levels of triacylglycerols in both groups, with additional reductions of very-long-chain ceramides (>C22 acyl chains) in the PD+E group. During the exercise test, sphingomyelins and ceramides increased in both groups, but only PD+E resulted in improved clearance of these bioactive lipids from circulation during recovery. The specific lipid response during recovery from exercise was associated with improvements in fat oxidation during exercise.

**Conclusions/interpretation:**

Concurrent training during PD-induced weight loss improves cardiometabolic fitness and is associated with an enhanced ability to clear bioactive sphingomyelins and ceramides from the circulation in the recovery phase after an exercise test. The improved lipid response during recovery and its association with the capacity for fat oxidation suggest that concurrent training during PD-induced weight loss may augment lipid handling beyond diet-induced changes, although causal mechanisms cannot be established.

**Graphical Abstract:**

**Figure:**
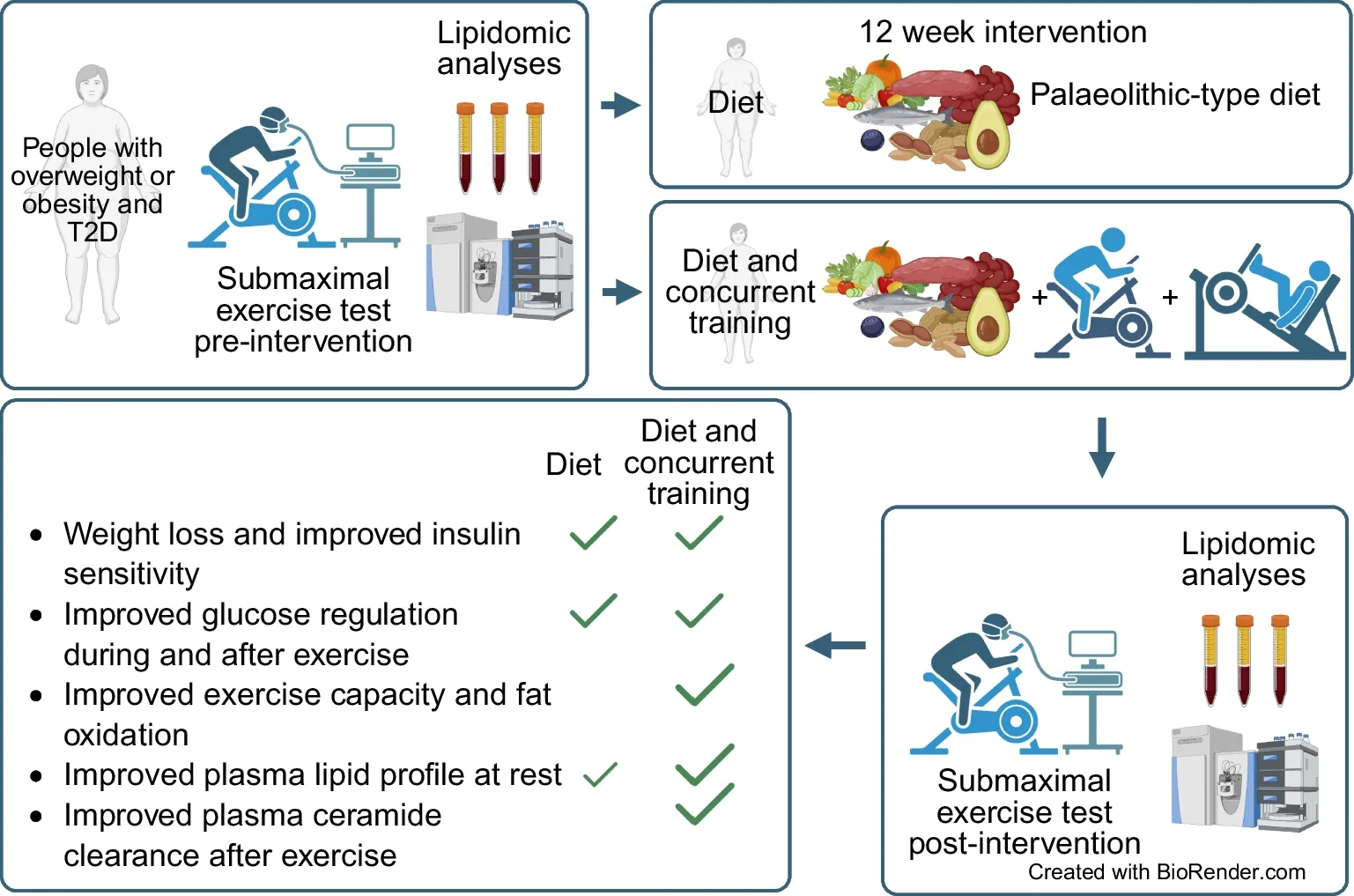

**Supplementary Information:**

The online version of this article (https://doi.org/10.1007/s00125-026-06820-4) contains peer-reviewed but unedited supplementary material.

**Figure:**
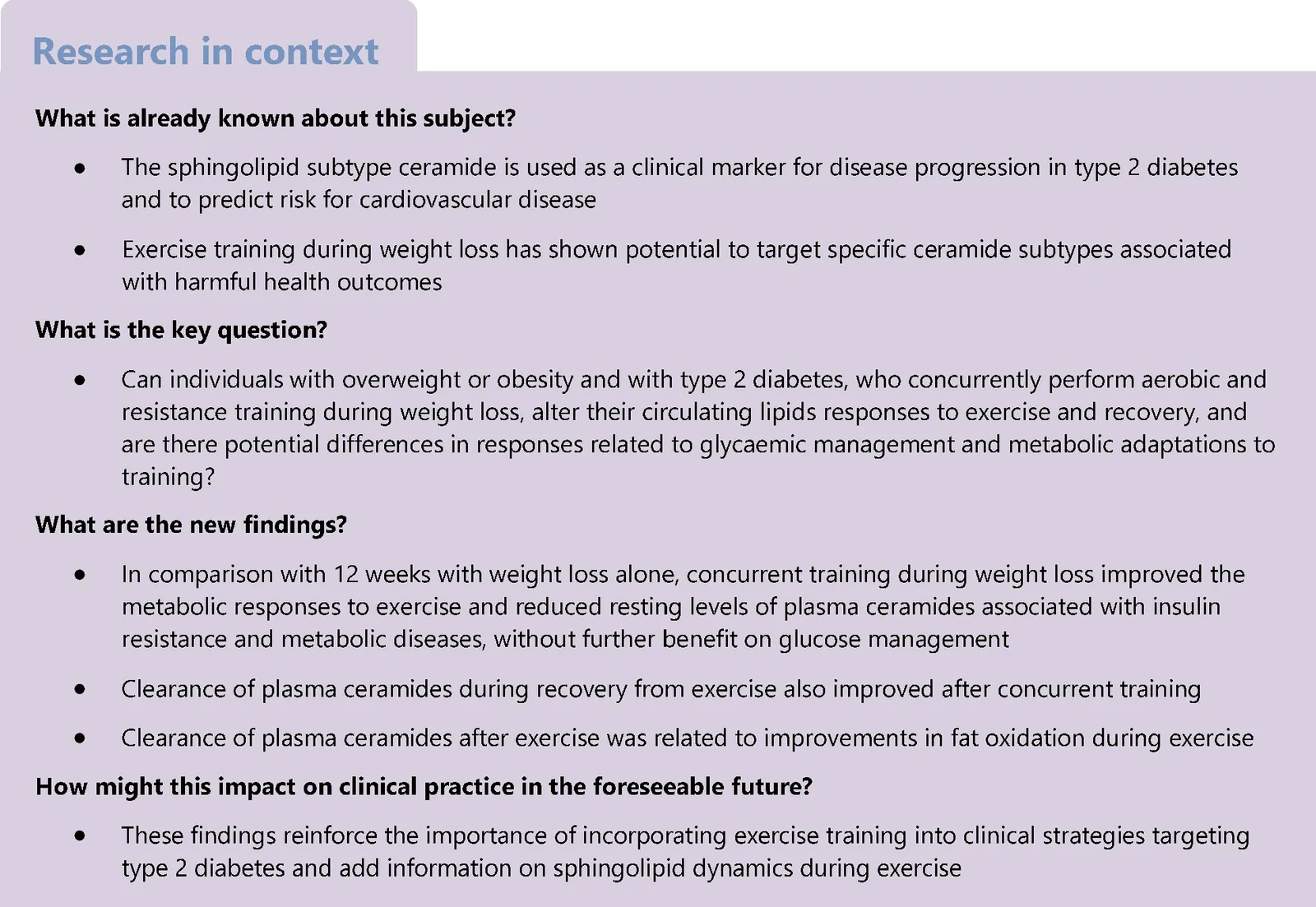

## Introduction

Lifestyle interventions that induce and maintain weight loss can improve peripheral insulin sensitivity and serve as a first-line strategy for type 2 diabetes management and remission, as defined as a non-diabetic glucose management in the absence of glucose-lowering drugs [1, 2]. The responsible mechanisms have not been elucidated but reducing lipid storage in key organs, such as the liver, muscle and pancreas, during weight loss is considered crucial [2, 3]. Paradoxically, exercise training during weight loss can counteract liver and skeletal muscle lipid depletion while improving glucose management [3]. A probable explanation is that exercise training improves lipid metabolism and reduces the accumulation of bioactive lipid species that interfere with insulin sensitivity [4–7].

Among bioactive lipids, sphingolipids play a significant role in cellular signalling and metabolic control. Ceramides and sphingomyelins are the major sphingolipid subtypes, and ceramides have been directly linked to insulin resistance [8, 9]. Circulating ceramides with specific acyl groups (C16, C18 and C24:1) have been shown to predict cardiometabolic outcomes beyond traditional markers such as cholesterol [10], and their reduction following weight loss has been shown to predict type 2 diabetes remission [11]. In contrast, sphingomyelins have gained less attention, and some studies have found them not to be associated with insulin resistance [12], whereas other studies have shown a negative association between certain sphingomyelins and diabetes risk [13] and health outcomes in obesity [14]. Still, reported changes in sphingomyelin levels in individuals with type 2 diabetes imply an important role in sphingolipid metabolism [15].

Although exercise training is considered effective for improving metabolic function and can affect lipids in storage and circulation, only a few studies have assessed changes in sphingolipids in individuals with type 2 diabetes in conjunction with exercise [13, 16, 17] and it is not known whether a lifestyle intervention, including diet-induced weight loss and exercise training, can influence sphingolipid responses to exercise.

To investigate this, we used a 12 week lifestyle intervention wherein participants with overweight or obesity, who were also living with type 2 diabetes, were randomised to a palaeolithic-type diet (PD) with or without concurrent training (aerobic and resistance training combined) three times weekly (PD+E). Lipidome analyses were conducted at rest, immediately after a standardised exercise test, and during recovery. The diet is central in this trial. The PD represents a whole-food dietary pattern characterised by lower carbohydrate intake and exclusion of grains, dairy and refined foods, with relatively higher intakes of vegetables, fruit, nuts, eggs, fish and lean meat. PD has been studied in type 2 diabetes and has been shown to produce clinically meaningful improvements in adiposity and metabolic risk factors, including glycaemic management and circulating lipids, often alongside spontaneous energy restriction when consumed ad libitum [18]. From a mechanistic perspective, PD-induced weight loss and reduced carbohydrate availability are expected to lower circulating triacylglycerols and alter lipid flux, factors closely linked to sphingolipid metabolism [6]. We therefore used PD as a standardised, clinically relevant weight-loss background to test whether concurrent training provides additive changes in circulating lipid dynamics during exercise and recovery in individuals with type 2 diabetes. We hypothesised that 12 weeks of concurrent training during PD-induced weight loss would improve metabolic fitness and responsiveness to exercise and that improvements would be accompanied by altered circulating lipid responses during exercise and recovery.

## Methods

### Study design and relationship to prior reports

**Fig. 1:**
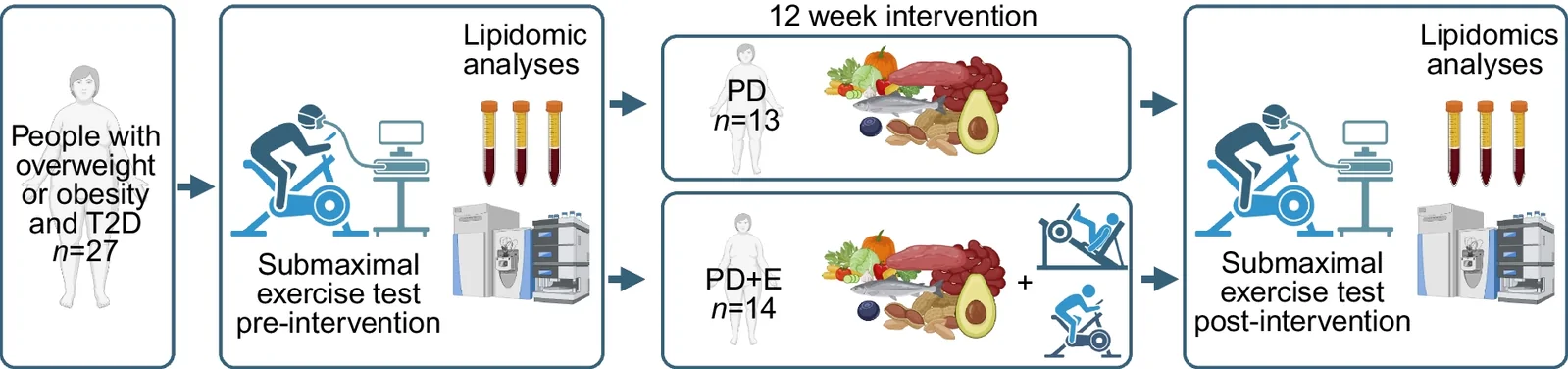
Study design. Participants with overweight or obesity and with type 2 diabetes were randomly assigned to 12 weeks of intervention with PD or PD+E. Before and after the intervention, venous blood samples were collected at rest, directly after an exercise test, and after 30 min of recovery (further illustrated in Fig. 2a). The figure was created with BioRender.com

This study is based on an intervention wherein individuals with type 2 diabetes were randomly assigned to 12 weeks of PD or PD+E in which PD was combined with three weekly sessions of supervised concurrent training (aerobic and resistance training combined). A schematic of the study design is presented in Fig. 1. Results from this randomised controlled trial have previously been published: a detailed description of the study cohort, intervention, body composition, diet, and glucose management are reported in [18]; liver and skeletal muscle fat storage in [3]; cardiac parameters in [19]; and metabolomic and lipidomic analyses of plasma and skeletal muscle in fasting samples at rest in [6]. The study was registered as a clinical trial (ClinicalTrials.gov registration no. NCT01513798). The original study cohort included 29 participants. Of them, 27 participants performed the exercise tests and are included in this manuscript. The lipidomic analysis of plasma samples obtained during the exercise test was performed later. Due to samples being sent for other analyses, a full set of samples from 21 participants were included in the analysis. In this study, we focused on the physiological responses during the exercise test and the lipids that discriminated most between groups at rest and in response to exercise and recovery.

### Participants’ characteristics and lifestyle intervention

The study was conducted according to the guidelines of the Declaration of Helsinki and approved by the Ethics Committee of Umeå University (Dnr2011-294-31 M). Informed consent was obtained from all participants involved in the study.

The individuals included were men and postmenopausal women with overweight or obesity who reported less than 30 min of moderate physical activity five times per week, did not engage in regular resistance training and had their type 2 diabetes diagnosis for more than 1 year. Participants’ characteristics are presented in Table 1. Sex and gender were self-reported by participants and were considered during randomisation to keep the treatment groups balanced. The study was not powered for separate sex/gender-stratified analyses. The PD was based on fish, lean meat, nuts and vegetables and can be regarded as a diet high in mono- and polyunsaturated fatty acids and protein and low in saturated fatty acids and carbohydrates. The diet was consumed ad libitum and resulted in an energy deficit in both groups. All participants received standard diabetes care instructions involving 30 min of daily moderate-intensity exercise (brisk walking, light cycling, etc.). Energy expenditure was monitored using a combined accelerometer and heart rate monitor (Actiheart; CamNtech, Cambridge, UK). Physical activity was also logged by the participants and communicated to the investigators during the study period to achieve compliance with the study design and avoid interference with the exercise intervention. The participants were also introduced to the PD, had a cooking class and got access to recipes. Throughout the intervention, they were supported by a dietitian. For PD+E, three supervised training sessions were performed each week at the Sports Medicine Unit at Umeå University. A week's training included three 1 h sessions of continuous or interval moderate-intensity exercise, high-intensity interval training or sprint interval training on a cycle ergometer combined with resistance exercises. The training programme followed a linear progressive design and a whole-body approach to improve cardiometabolic fitness (${\dot{V}\mathrm{O}}_{2\mathrm{m}\mathrm{a}\mathrm{x}}$ and metabolic function), muscular endurance and strength and to preserve lean mass during weight loss. Resistance exercises comprised two or three sets of 10–15 repetitions (performed at ~75–68% of their one-repetition maximum, when applicable). All sessions were supervised by certified strength and conditioning coaches, and participants' adherence exceeded 85%. The complete training programme is provided in electronic supplementary material (ESM) Methods.

**Table 1:** Participants’ characteristics and measures of glucose, insulin and insulin sensitivity

Characteristic	PD		PD+E		*n* PD/PD+E	Baseline comparisons (*p* value)
	Pre	Post	Pre	Post		
Sex, *n* male/*n* female	4/9		5/9		13/14	
Age, years	59±7		61±6		13/14	0.19
BMI, kg/m^2^	31.2±3.4	28.4±2.6***	32.2±3.9	29.6±4.0***	13/14	0.55
Fasting glucose, mmol/l	7.7±1.0	6.7±1.5*	9.8±2.7	7.4±2.1**	12/14	0.03
Fasting insulin, pmol/l	158±63	90±35**	119±59	74±26**	12/14	0.10
HbA_1c_, mmol/mol	54±7	43±6**	59±14	45±13**	13/14	0.25
HbA_1c_, %	7.1±2.8	6.1±2.7**	7.5±3.4	6.3±3.4**		
Revised QUICKI	0.22±0.02	0.25±0.04**	0.22±0.03	0.26±0.04***	12/12	0.88
HOMA-IR	7.5±2.5	4.0±2.1***	6.8±2.8	3.6±2.1***	12/13	0.50
Peripheral insulin sensitivity^a^	0.041±0.018	0.082±0.037**	0.053±0.021	0.072±0.024*	12/12	0.15

Results are presented as means ± SD

A paired Student’s *t* test assessed differences between baseline and after 12 weeks of intervention and an unpaired Student’s *t* test assessed differences between groups at baseline: *p<0.05, **p<0.01, ***p<0.001

^a^Rate of disappearance, mg kg^−1^ min^−1^ [basal insulin]^−1^ during a hyperinsulinaemic–euglycaemic clamp

### Exercise testing and blood sampling

**Fig. 2:**
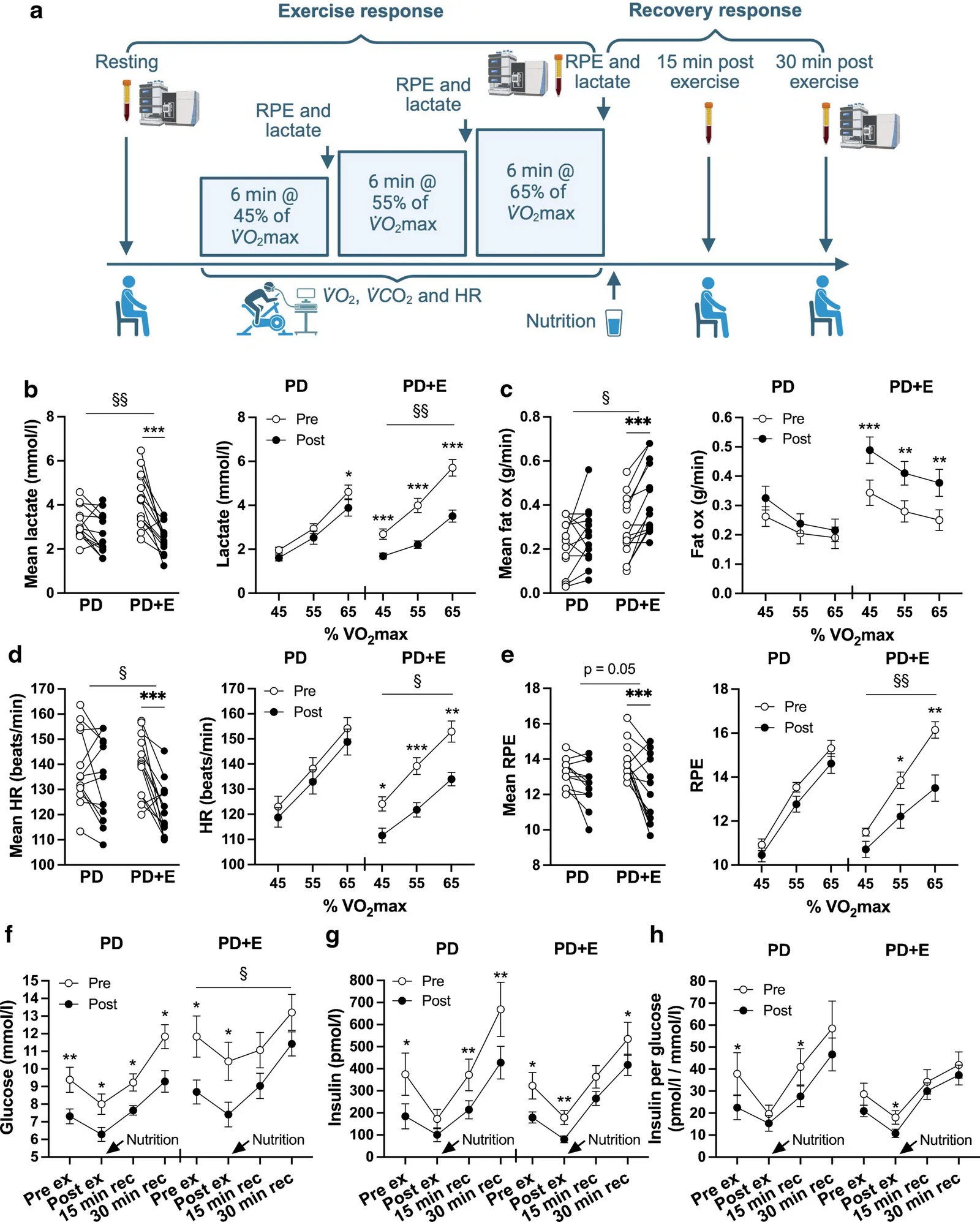
Physiological responses during submaximal exercise testing. (**a**) The participants exercised on a cycle ergometer at three different work rates. ${\dot{V}\mathrm{O}}_{2}$, ${\dot{V}\mathrm{C}\mathrm{O}}_{2}$ and heart rate were measured continuously during exercise. Lactate and RPE were collected during rest periods. A drink containing 1 g/kg body weight carbohydrates and 0.21 g/kg body weight protein was ingested within 1 min after cessation of exercise. Blood samples for MS-based lipidomic analysis were collected at rest before exercise, immediately after exercise and after 30 min of recovery. Glucose and insulin were analysed in samples collected at the same time points and 15 min after exercise. (**b**–**e**) Lactate (**b**), fat oxidation (**c**), heart rate (**d**) and RPE (**e**) during submaximal exercise. Individual mean values and statistical differences between groups pre- to post-intervention are shown; within-group responses to different work rates (effect of the intensity of exercise) are also displayed. *n*=13 (PD) or 14 (PD+E). (**f**–**h**) Glucose (**f**), insulin (**g**) and the insulin/glucose ratio (**h**) during exercise and recovery are shown, with statistical comparisons made within groups. *n*=12 (PD) or 13 (PD+E). A repeated measures two-way ANOVA was used to assess differences between groups pre- to post-intervention and within groups at different time points. ^§^*p*<0.05, ^§§^*p*<0.01 for interaction effect of the intervention (pre- to post-intervention and group or time). As a post hoc test, Šídák's multiple comparisons test assessed differences at different time points: *p<0.05, **p<0.01, ***p<0.001. Data are presented as mean ± SD. ex, exercise; HR, heart rate; ox, oxidation; rec, recovery. Figure 2a was created with BioRender.com

Before and after the lifestyle intervention, the physiological responses during submaximal exercise were assessed. The participant reported to the laboratory in the afternoon after a 2 h fast. First, a catheter was inserted in the antecubital vein, and a blood sample was collected. Thereafter, the participant mounted a cycle ergometer (Monark 894; Monark Exercise) and cycled for 6 min each at work rates corresponding to 45, 55 and 65% of ${\dot{V}\mathrm{O}}_{2\mathrm{m}\mathrm{a}\mathrm{x}}$. ${\dot{V}\mathrm{O}}_{2}$ and ${\dot{V}\mathrm{C}\mathrm{O}}_{2}$ (Jaeger Oxycon Pro system; Erich Jaeger, Hoechberg, Germany) and heart rate (Polar Electro OY; Kempele, Finland) were measured continuously. Capillary blood was collected after each work period and analysed for lactate (YSI 2300 STAT Plus; Yellow Springs, OH, USA). Ratings of perceived exertion (RPE) were assessed using the Borg scale [20]. Substrate metabolism was calculated using the equation proposed by Jeukendrup and Wallis [21]. The same absolute work rates were used before and after the intervention. Immediately after completing the last stage, a venous blood sample was collected and the participant ingested a non-commercial beverage provided by a local dairy producer (blended and donated by Norrmejerier Dairy Cooperative, Umeå, Sweden) in an amount corresponding to 1.0 g of carbohydrates (maltodextrin) and 0.2 g of milk proteins (80% casein, 20% whey) per kg body weight. The same absolute amounts were used before and after the intervention. Further blood samples were obtained at 15 and 30 min into recovery. After collection, all blood samples were put on ice, centrifuged and the plasma was transferred to new tubes and stored at −80°C. A schematic of the exercise test can be found in Fig. 2a.

### Glucose, insulin and insulin sensitivity

Plasma glucose was analysed on an Abbott Architect system, using Abbott's hexokinase reagents. Serum insulin was analysed using a sandwich immunosorbent assay on a Cobas E601 immunology analyser (Roche Diagnostics). HbA_1c_ was analysed immunologically on a Cobas c501 (Roche Diagnostics) using Tina-Quant reagents. Data for glucose, insulin and HbA_1c_ presented in Table 1 were collected in a fasted state during rest pre- and post-intervention on a separate occasion. HOMA-IR was calculated as follows: (fasting glucose × fasting insulin) / 22.5. The revised quantitative insulin sensitivity check index (Revised QUICKI) was calculated as follows: 1 / (log_10_ fasting glucose + log_10_ fasting insulin + log_10_ NEFA). Peripheral insulin sensitivity assessed with a hyperinsulinaemic–euglycaemic clamp was calculated as follows: rate of disappearance = mg kg^−1^ min^−1^ basal insulin. Glucose and insulin were also collected during the exercise tests at the time points displayed in Fig. 2a.

### MS analysis

Plasma samples obtained before, during and after exercise tests were subjected to targeted lipidomic analysis via LC time-of-flight MS operating in positive mode (LC-TOF/MS; Agilent Technologies, Santa Clara, CA, USA). Sample preparation prior to analysis adhered to a modified protocol [22]. Detailed descriptions of the sample preparation and analytical procedures are available in [23]. The LC-MS data were processed using Agilent MassHunter Profinder version B.08.00 (Agilent Technologies). Lipids are annotated according to the standardised lipid nomenclature established by the Lipid Maps Lipidomics Gateway (lipidmaps.org). This annotation includes the lipid class, the total number of carbon atoms in the attached fatty acids, and the total number of double bonds.

### Statistical analysis

Statistical analyses were conducted using SIMCA 18.0 (Sartorius, Umeå, Sweden) and GraphPad Prism v 9.5.0 (GraphPad Software, La Jolla, CA, USA). The lipid screening identified 225 distinct lipids, of which some showed high intra- and inter-individual variability. Considering the limited number of participants in the analysis, a univariate statistical approach is expected to falsely identify some lipids as significantly altered between groups or time points. However, given our exploratory approach, we prioritised sensitivity to detect biologically relevant lipid changes, accepting a potential increase in false-positives as a trade-off. We therefore first applied a multivariate statistical approach to investigate which lipids contributed most to the variability in the dataset and proceeded with univariate statistics to show changes in selected lipids and lipid classes. For an initial inspection of data, principal component analysis (PCA) was employed to identify groupings, outliers and trends within the datasets. Subsequently, orthogonal partial least squares (OPLS) analysis was applied to compare lipid signature, pre- and post-intervention (rested values), as well as relative to the acute bout of exercise (rested vs post-exercise) and during recovery (post-exercise vs 30 min into recovery). Identified lipid signatures were selected for further statistical analysis based on their variable importance in projection (VIP, threshold of 1), derived from the OPLS model. These metabolites were subsequently validated at the univariate level, with significance set at a Bonferroni-adjusted *p* value using a 95% CI. Multivariate models were validated through the ANOVA of cross-validated OPLS scores (CV-ANOVA) to confirm statistical significance and reduce the risk of overfitting. Parametric tests were selected based on normality assessments, and significant jack-knifing-based confidence intervals derived from the OPLS models. Comparisons between means were done with a Student’s *t* test and differences between groups pre- to post-intervention were analysed with repeated measures two-way ANOVA with Šídák's multiple comparisons test for post hoc analysis. Where data were missing, a mixed-effect model was used with Tukey’s multiple comparison test. Differences between time points within groups were analysed with repeated measures one-way ANOVA with Tukey’s multiple comparisons test for post hoc analysis. When possible, the Geisser–Greenhouse correction was applied to correct for uneven variance. Both univariate and multivariate lipid levels originate from metabolomics analyses and should therefore be considered exploratory.

## Results

### 12 weeks of PD resulted in weight loss and improved insulin sensitivity regardless of exercise intervention

Participants’ characteristics and clinical outcomes are presented in Table 1, and data for energy balance and macronutrient dietary intake are presented in ESM Table 1. These data have previously been reported for the original study cohort [3, 19]. In all, we report similar improvements in glucose management and weight loss in both groups. Compliance with the diet intervention was similar in both groups, resulting in an energy deficit, mainly through reduced intake of carbohydrates and saturated fat, and an increase in protein intake.

### Concurrent training during PD-induced weight loss improved exercise tolerance but did not affect glucose management during and after exercise

The physiological responses during the exercise test and the subsequent responses to a nutritional load were studied pre- and post-intervention. A schematic of measurements and time points for blood sampling is shown in Fig. 2a. At exercise intensities between 45–65% of ${\dot{V}\mathrm{O}}_{2\mathrm{m}\mathrm{a}\mathrm{x}}$, the PD+E group showed reduced mean lactate levels, increased fat oxidation, reduced heart rate and reduced RPE on the Borg RPE-scale compared with pre-intervention. With the exclusion of fat oxidation, an effect of exercise intensity was evident for all measures (Fig. 2b–e). PD improved none of these measures. To investigate whether the intervention benefitted skeletal muscle glucose uptake after exercise, we challenged our participants with a bolus dose of glucose and protein, which was consumed within 1 min after exercise. As expected, glucose and insulin levels, as well as the insulin/glucose ratio, decreased during exercise and increased after the nutritional load and reached values above baseline (Fig. 2f–h). However, these values improved similarly in both groups, indicating no differences in glucose regulation and insulin sensitivity in this setting.

### Concurrent training during PD-induced weight loss reduced sphingolipids beyond the effect of diet-induced weight loss

**Fig. 3:**
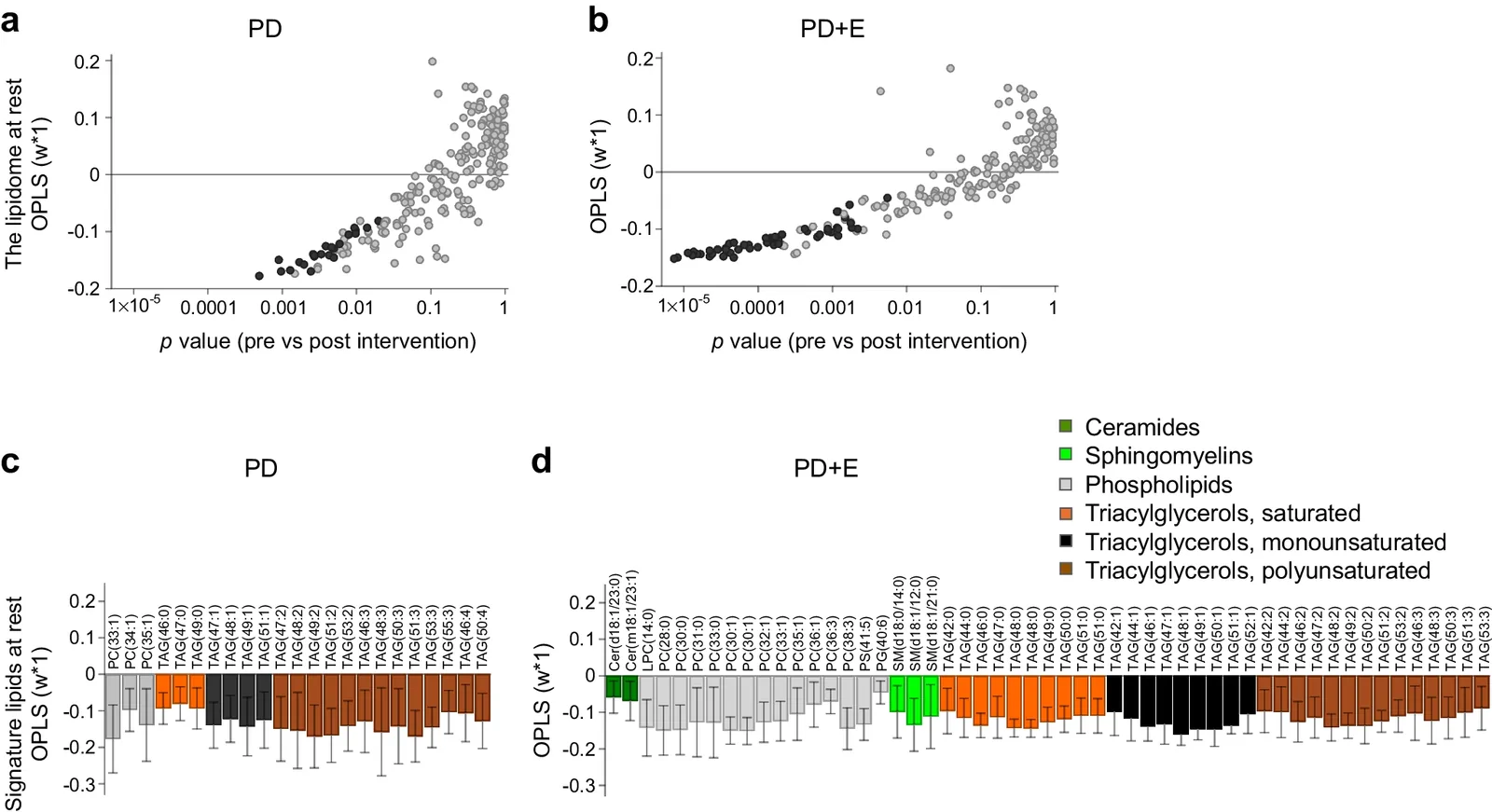
Changes in plasma lipids at rest in response to the lifestyle intervention (*n*=10 [PD] or 11 [PD+E]). (**a**, **b**) OPLS models illustrate the effect of the intervention on the plasma lipidome in participants assigned to PD (**a**) or PD+E (**b**) for 12 weeks. Each circle represents a lipid species, with black circles indicating significantly altered lipids, defined by VIP> 1 and significant correlation coefficients (based on jack-knifing confidence intervals). The *y*-axis shows the OPLS predictive component (w*1), and the *x*-axis shows univariate *p* values comparing pre- and post-intervention lipid levels. (**a**) CV-ANOVA *p*=0.04; (**b**) CV-ANOVA *p*=0.0002. (**c**, **d**) Significantly altered lipid species in PD (**c**) and PD+E (**d**), respectively, based on OPLS loadings and univariate significance of *p*<0.05. (**c**) CV-ANOVA *p*=0.04; (**d**) CV-ANOVA *p*=0.0002. Data are presented as mean ± SD. Cer, ceramide; LPC, lysophosphatidylcholine; PC, phosphatidylcholines; PG, phosphatidylglycerol; PS, phosphatidylserine; SM, sphingomyelin; TAG, triacylglycerol

Using MS-based lipidomics, we identified 225 distinct lipids in plasma samples collected at rest, post-exercise and during recovery. Initial inspection via PCA did not reveal significant outliers (ESM Fig. 1). At rest, the lifestyle intervention led to significant alterations in lipid profiles for both groups, with more pronounced changes in the PD+E group than in the PD group (OPLS, CV-ANOVA; PD, *p*=0.04; PD+E, *p*=0.0002) (Fig. 3a, b). Levels of phosphatidylcholines and specific triacylglycerols with low degrees of saturation (total number of double bonds for all acyl chains >4) decreased in both groups, with additional decreases of triacylglycerols with higher saturation in the exercise group (Fig. 3c, d). Notably, PD+E further lowered levels of sphingolipids (i.e. very-long-chain ceramides [>C22 acyl chains] such as C23:0 and C23:1) and specific sphingomyelins (such as C14:0, C12:0 and C21:0), which was not evident in PD (Fig. 3c, d). Total resting levels of triacylglycerols and sphingolipids according to saturation and class, and volcano plots showing changes of specific triacylglycerols and sphingolipids, are available in ESM Fig. 2, with corresponding *p* values and fold changes available in ESM Table 2.

### Concurrent training during PD-induced weight loss improved sphingolipid clearance during recovery from exercise

**Fig. 4:**
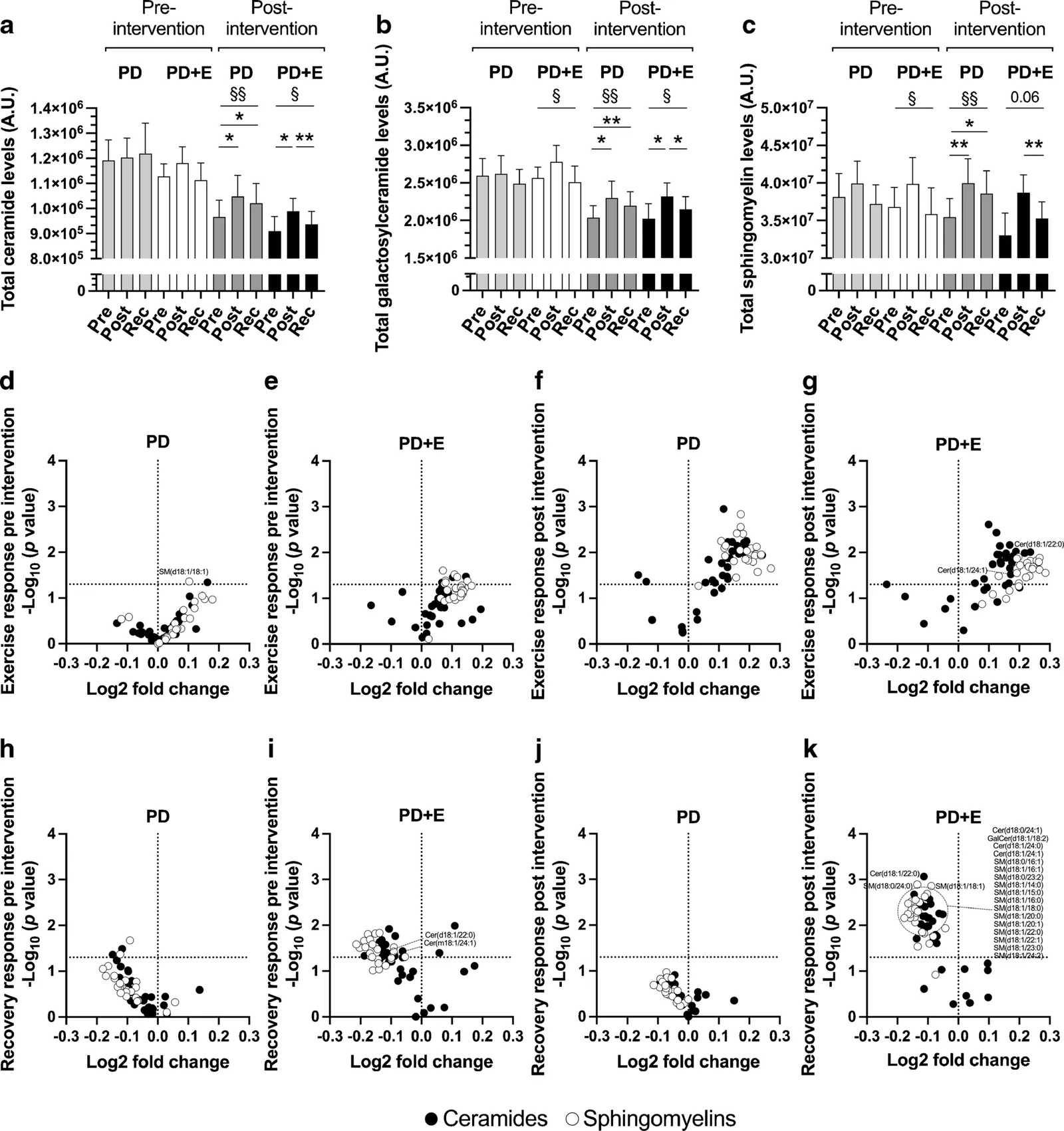
Changes in plasma sphingolipids in response to exercise and recovery before and after the intervention. (**a**–**c**) Total levels of ceramides (**a**, *n*=26 species), galactosylceramides (**b**, *n*=8 species) and sphingomyelins (**c**, *n*=26 species) in response to exercise and after 30 min of recovery according to lipid classes. *n*=10 (PD) or 11 (PD+E). Repeated measures one-way ANOVA assessed the effect of time within groups: ^§^*p*<0.05, ^§§^*p*<0.01. As a post hoc test, Tukey’s multiple comparisons test assessed differences between time points: *p<0.05, **p<0.01. Data are presented as mean ± SD. (**d**–**k**) Volcano plots show each sphingolipid’s response to exercise (post-exercise to pre-exercise) and recovery (30 min recovery to post-exercise). *n*=10 (PD) or 11 (PD+E). In volcano plots, the *x*-axis shows the magnitude and direction of change, and the *y*-axis shows the significance of change. The horizontal dotted line at 1.3 in volcano plots represents a significant change of *p*=0.05. In (**h**) two outliers are not shown; please see ESM Table 2 for more information. Annotated lipids are those identified as different between time points according to the OPLS analysis and significantly changed according to *t* tests (*p*<0.05). A.U., arbitrary units; Rec, recovery

To investigate alterations in plasma lipids in response to exercise and recovery, we performed a multivariate analysis of lipid profiles from samples collected at rest, immediately after exercise and 30 min into recovery. Using an OPLS model, we found that the intervention significantly affected sphingolipids and one polyunsaturated triacylglycerol. The most noteworthy changes distinguishing the groups occurred during the recovery phase (post-exercise vs 30 min into recovery) in the PD+E group. An overview of changes in these lipids across all phases is presented in ESM Fig. 3. A subsequent univariate analysis was performed to further explore the dynamic changes of sphingolipids during exercise and recovery. Total sphingolipid levels by class are shown in Fig. 4a–c, while changes in specific sphingolipids are shown in Fig. 4d–k. Before the intervention, total levels of ceramides, galactosylceramides and sphingomyelins showed minor alterations in response to exercise and recovery. The intervention markedly changed these responses in both groups. During exercise, several of the C14–C20 ceramides decreased (see ESM Table 2 for specific *p* values), while VLC ceramides increased in both groups after the intervention (Fig. 4f, g). After 30 min of recovery, differences emerged between the groups. In the PD group, plasma sphingolipid clearance appeared to be suppressed slightly after the intervention, regardless of lipid class (Fig. 4j). Contrastingly, the PD+E group showed effective clearance of almost all sphingomyelins, galactosylceramides and VLC ceramides from circulation after the intervention (Fig. 4k). Notably, these lipid species increased during exercise. C14–C20 ceramides that did not increase during exercise appeared less affected during recovery. The specific sphingolipids that showed significant changes across both exercise and recovery according to both OPLS modelling and *t* tests are annotated in Fig. 4d–k. In this manuscript, ‘clearance’ refers operationally to the reduction in circulating lipid concentrations during recovery after exercise, rather than direct measurement of tissue uptake, degradation or lipid flux.

### Changes in lipid response to recovery correlated with improved fat oxidation during exercise

**Fig. 5:**
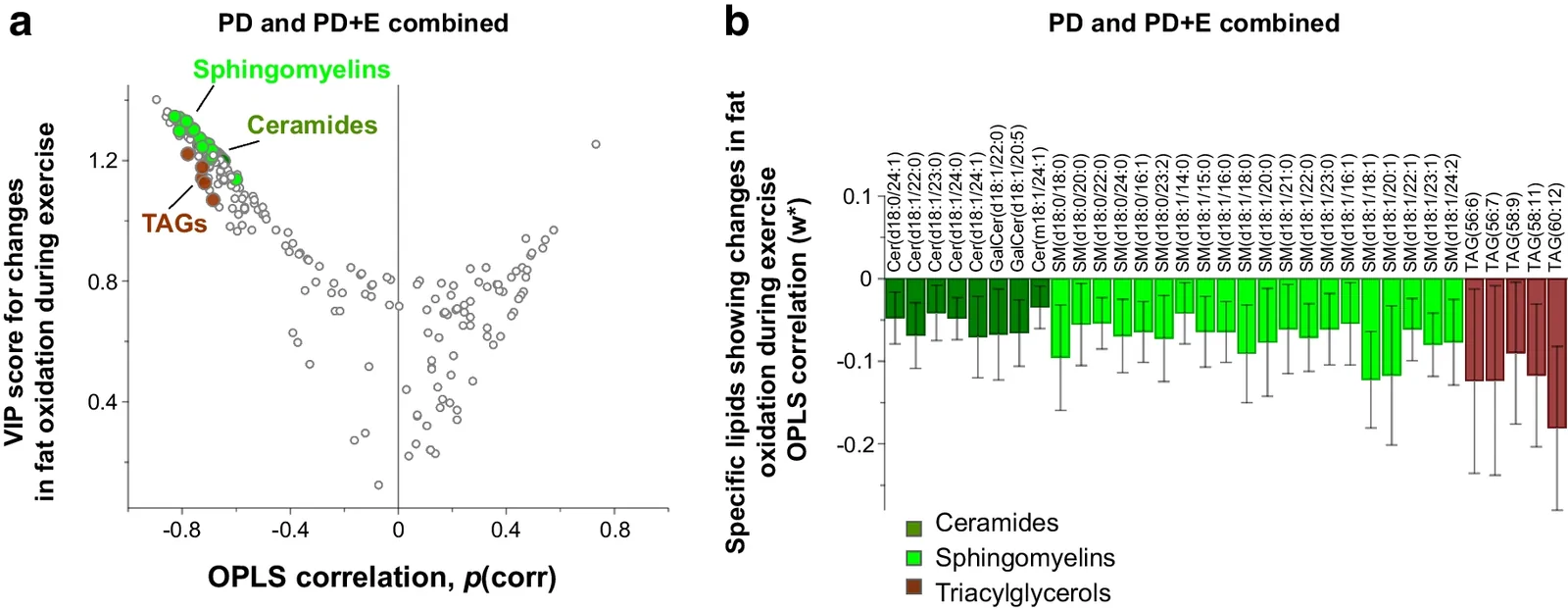
Lipid responses during recovery are associated with improvements in fat oxidation during exercise. (**a**) Multivariate correlation between lipid responses to recovery (exercise vs 30 min post-exercise) and Δ fat oxidation during exercise (post- to pre-intervention) based on an OPLS model. The *y*-axis represents the VIP score from the OPLS model. The *x*-axis shows the multivariate correlation of each lipid’s response to fat oxidation. Highlighted lipids have a combined VIP>1 and are multivariately significant based on jack-knifing testing. (**b**) Changes of specific sphingolipids and one polyunsaturated triacylglycerol that correlate with Δ fat oxidation during exercise. CV-ANOVA *p*=0.01. A negative value indicates that lowering these lipids is linked with improved fat oxidation during exercise. Identical lipids are annotated in (**a**) and (**b**). Model parameters of OPLS components are: 1P+1O describes systematic variation (R2: 66%); and predictive variation (Q2: 45%). Data are presented as mean ± SD. PD and PD+E combined (*n*=21). Cer, ceramide; SM, sphingomyelin; TAG, triacylglycerol

In search of a physiological outcome that could explain the observed changes in lipid response during recovery, we performed an additional OPLS analysis correlating changes in lipid status in response to recovery with changes in physiological outcomes of the intervention (Δ post- to pre-intervention). Across both groups, a significant correlation was observed between changes in fat oxidation during exercise and several ceramides, sphingomyelins and polyunsaturated fatty acid-containing triacylglycerols in response to recovery (OPLS correlation, CV-ANOVA *p*=0.01) (Fig. 5a). The specific lipids that significantly correlate with changes in fat oxidation are highlighted in Fig. 5b.

## Discussion

This study suggests additional benefits for cardiometabolic capacity and circulating lipids in individuals with overweight or obesity, who were living with type 2 diabetes and who performed concurrent training during PD-induced weight loss. While both PD and PD+E groups achieved comparable weight loss and improvements in glycaemic management, concurrent training during weight loss was associated with a more favourable resting lipid profile and a recovery-phase lipid response characterised by a greater reduction in circulating ceramides and sphingomyelins following exercise. Notably, the differences in total sphingolipid levels observed after exercise were not apparent at rest, suggesting that the primary effect of concurrent training during weight loss relates to lipid handling during metabolic stress and recovery rather than baseline concentrations. Collectively, these findings suggest that, within a shared dietary weight-loss context, concurrent training may augment aspects of lipid metabolism beyond those achieved by diet alone.

### Can exercise training reduce harmful sphingolipids in circulation?

Although total resting levels of plasma ceramides, galactosylceramides or sphingomyelins did not differ between groups post-intervention, the selective reduction in VLC ceramides in the PD+E group suggests nuanced changes in lipid metabolism. Plasma ceramides are established risk markers of type 2 diabetes, cardiovascular diseases and mortality [10, 11, 24, 25]. Notably, the VLC ceramide C24:1 is a potential biomarker for unhealthy metabolic outcomes in obesity [26]. Our findings support the growing evidence that exercise interventions can reduce circulating and skeletal muscle ceramides in parallel with improved glucose management [5, 27]. Indeed, the observation that PD+E reduced resting levels of VLC ceramides and enhanced their clearance after exercise is of particular significance since they are independent predictors of cardiovascular events [28–30] and have been implicated in obesity-related inflammation and insulin resistance [31]. Ceramides are also implicated in cardiovascular disease and contribute to lipoprotein aggregation and atherosclerotic plaque formation [32], though the underlying mechanism remains unclear.

Ceramide levels in the bloodstream are governed by a tightly regulated interplay between their synthesis, transport and degradation. However, the mechanism underlying the regulation of VLC ceramides remains poorly understood [33]. The preferential reduction in VLC ceramides observed post-intervention may reflect exercise-induced adaptations in ceramide trafficking and metabolism. VLC ceramides are poor substrates for the ceramide transfer protein (CERT), which primarily transports ceramides with acyl chains up to C20 [34]. As such, VLC species rely on vesicular transport or alternative non-CERT transport mechanisms for cellular trafficking, either by promoting vesicular lipid export or increasing intracellular processing [33]. Recent evidence suggests that VLC ceramides are selectively packaged into extracellular vesicles under lipotoxic conditions, a process that may be amplified by exercise-induced improvements in metabolic flexibility [35]. Thus, the observed reduction in circulating VLC ceramides during recovery may reflect enhanced transport and turnover capacity.

PD+E also reduced specific circulating sphingomyelins at rest and during recovery from exercise, and these changes were associated with improved fat oxidation. Sphingomyelins are both a substrate and a product for ceramides produced in the sphingomyelinase pathway [36] and are a substrate in the salvage pathway [37]. Consequently, even if sphingomyelins themselves are not as strongly related to insulin resistance or implicated in mitochondrial dysfunction as ceramides [12], their reduction may signal enhanced ceramide clearance at the cellular level, potentially lowering cardiometabolic risk [38].

### Sphingolipid response to exercise and recovery

The rise in circulating ceramides during exercise may be attributed to increased catecholamine levels that stimulate hormone-sensitive lipase and adipose triglyceride lipase, leading to an enhanced release of fatty acids and potentially ceramides. Previous reports suggest varying responses based on fitness levels and metabolic disorders. For example, Helge et al found that endurance athletes exhibited greater increases in skeletal muscle ceramides and sphingomyelin levels after prolonged exercise than in untrained individuals [17], while Bergman et al observed a reduced ceramide response in endurance-trained athletes compared with individuals with type 2 diabetes [16]. Plasma levels of sphingolipids from the latter study were reported separately, and athletes were found to have a greater increase in C16–C24 ceramides during exercise than individuals with type 2 diabetes, with similar decreases observed during recovery [13]. In contrast, C14–C24 sphingomyelins showed small decreases during exercise and were further reduced after recovery, with larger reductions observed in individuals with type 2 diabetes than in athletes [13]. Contrastingly, we observed that both groups increased plasma sphingolipids during exercise, but only PD+E effectively reduced these lipids during recovery from exercise after the intervention. It is therefore plausible that adaptations in metabolic pathways following exercise training influence the release and/or uptake of ceramides by skeletal muscle or the liver during exercise and recovery. It is unclear which organs are responsible for regulating circulating ceramides post-exercise, and whether this regulation is influenced by exercise training independently of reductions in ectopic lipid storage through a hypoenergetic diet. The liver, a known source of circulating ceramides [39, 40], is a likely candidate, though its role in ceramide clearance remains poorly understood.

### Diet effects vs exercise effects

Because both groups adhered to the PD, several lipid changes observed at rest likely reflect PD-induced weight loss and altered substrate availability, including the marked reduction in circulating triacylglycerols in both groups. Against this shared dietary background, the main additional effect associated with concurrent training was the more favourable sphingolipid profile. Accordingly, our conclusions are limited to the context of PD-induced weight loss, and the present design does not allow complete separation of diet-specific effects from those of exercise training. However, it should be noted that previous research suggests that dietary interventions, including low-fat [41] and Mediterranean diets [42], do not affect ceramide levels unless they specifically reduce the intake of palmitic acid, which is the primary precursor of ceramide [43]. Future studies addressing these questions would need to disentangle the effects of weight loss from those of exercise training, ideally using tracer-based approaches to map ceramide fluxes in tissues and circulation.

### Strengths and limitations

The design of this study reflects a real-world, feasible strategy for improving cardiometabolic health in type 2 diabetes management. The use of an individually standardised exercise test allowed us to amplify and capture metabolic signals and changes in the circulating lipidome not observable in a fasted state. However, plasma samples might therefore have been influenced by dietary intake. Furthermore, the participants also continued their medications during the intervention. The study design also has some limitations, including potential variations in self-reported physical activity and lack of an exercise-only arm. Because all participants received standard diabetes care advice to perform daily moderate-intensity physical activity, the present comparison tests the addition of supervised concurrent training to PD-induced weight loss and background physical activity advice, rather than exercise vs no exercise. Although Actiheart-derived physical activity energy expenditure data did not differ materially between groups, residual differences in activity intensity or pattern cannot be excluded. Both intervention groups followed the PD. Therefore, the independent effects of concurrent training cannot be separated from those of PD-induced weight loss. Similar effects on ectopic lipid stores, lipid metabolism and insulin sensitivity could have been achieved with exercise training alone, or the combined intervention produced additive effects or reached a ceiling effect. The findings should therefore be interpreted as effects of diet-induced weight loss with or without concurrent training, rather than effects of exercise training per se. Lastly, while the sample size provided sufficient power for many physiological measurements, the inherent intra- and inter-individual variability of lipid concentrations, as well as the limited understanding of the optimal time points for blood sampling, presented challenges to exploring the variability of lipid concentrations, especially in the subgroup analyses. In addition, the subgroup with complete lipidomic sampling was modest in size, and these findings should therefore be interpreted as hypothesis-generating. Although sex/gender was balanced between treatment groups, no sex/gender-stratified analyses were performed because of the limited sample size, particularly in the lipidomics subgroup. Potential sex/gender-specific differences in exercise adaptation, lipid metabolism and sphingolipid handling can therefore not be excluded, and the findings should be interpreted as applying to the mixed study population rather than being generalisable to all sexes/genders.

### Conclusions

This study suggests that concurrent training during PD-induced weight loss can provide additional benefits in older individuals with overweight or obesity who were living with type 2 diabetes by improving cardiometabolic fitness and lipid clearance 30 min after exercise, particularly for ceramides and sphingomyelins, which are associated with adverse metabolic outcomes. The observed association between changes in lipid clearance and changes in fat oxidation during exercise suggests that improved metabolic fitness may partly contribute to this effect.

## Supplementary Information

Below is the link to the electronic supplementary material.

ESM (PDF 674 KB)

ESM Table 2 (XLSX 43 KB)

ESM Methods (XLSX 20 KB)

## Data Availability

The datasets generated during and/or analysed during the current study are available from the corresponding author (elin.chorell@umu.se) upon reasonable request. The data are not publicly available because public sharing is restricted under the EU General Data Protection Regulation (GDPR) and applicable Swedish data-protection legislation.

## Abbreviations

CERT: Ceramide transfer protein
OPLS: Orthogonal partial least squares
PCA: Principal component analysis
PD: Palaeolithic-type diet
PD+E: Palaeolithic-type diet and exercise
RPE: Ratings of perceived exertion
VIP: Variable importance in projection
VLC ceramide: Very-long-chain ceramide (>C22 acyl chain length)
