# Supplementary material for: Concurrent training is associated with enhanced post-exercise ceramide clearance and fat oxidation capacity in individuals with type 2 diabetes following lifestyle intervention
Source: Diabetologia. 2026 Aug 5;69(11):3288–300. doi: 10.1007/s00125-026-06820-4 (PMC13627267; doi:10.1007/s00125-026-06820-4)
Supplement: Supplementary file 1 [file 125_2026_6820_MOESM1_ESM.pdf]

	Palaeolithic-type diet (PD) *n* = 13		Palaeolithic-type diet + exercise (PD+E) *n* = 14		*n* (PD/PD+E)	Baseline comparisons (*p* value)
	Pre	Post	Pre	Post		
**Energy balance**						
Energy intake (kJ/day)	8113 ± 2540	6230 ± 1757**	7586 ± 2092	5506 ± 2234***	12/13	0.58
Physical activity energy expenditure (kJ/day)	4950 ± 1059	4393 ± 1038	5050 ± 2335	4803 ± 2105	13/14	0.47
Physical activity energy expenditure (kJ/kg/day)	49.0 ± 11.3	52.3 ± 12.6	51.9 ± 20.9	54.4 ± 19.7	13/14	0.68
Resting energy expenditure (kJ/day)	6707 ± 858	6150 ± 770***	6933 ± 1431	6364 ± 1423*	12/11	0.64
Resting energy expenditure (kJ/kg/day)	71.6 ± 5.9	72.0 ± 5.9	71.6 ± 7.1	73.2 ± 11.7	12/11	0.90
Total energy expenditure (kJ/day)	12397 ± 1703	11661 ± 1494*	13180 ± 3694	123644 ± 3473	12/11	0.51
**Dietary intake**						
Protein (g/day)	84 ± 27	90 ± 26	88 ± 31	84 ± 31	12/13	0.70
Carbohydrate (g/day)	198 ± 68	116 ± 45***	182 ± 51	89 ± 38***	12/13	0.51
Total fat (g/day)	83 ± 28	68 ± 28	73 ± 29	64 ± 33	12/13	0.38
Protein (E%)	17 ± 3	25 ± 6***	19 ± 3	26 ± 4***	12/13	0.16
Carbohydrate (E%)	41 ± 5	32 ± 11**	41 ± 9	28 ± 10***	12/13	0.93
Total fat (E%)	39 ± 5	40 ± 12	35 ± 7	42 ± 11	12/13	0.24
Saturated fatty acids (g/day)	31 ± 12	15 ± 6**	28 ± 11	14 ± 7***	12/13	0.55
Monounsaturated fatty acids (g/day)	33 ± 11	35 ± 17	27 ± 12	32 ± 17	12/13	0.22
Polyunsaturated fatty acids (g/day)	12 ± 4	13 ± 6	11 ± 5	12 ± 7	12/13	0.43
Omega-3 fatty acids (g/day)	2.5 ± 1.3	2.8 ± 1.8	2.2 ± 1.2	2.6 ± 1.4	12/13	0.61
Omega-6 fatty acids (g/day)	10.8 ± 4.4	10.9 ± 5.8	9.6 ± 4.8	10.6 ± 6.2	12/13	0.52

ESM Table 1 Energy balance and dietary intake. Energy expenditure was measured with a combined heart rate and accelerometer (Actiheart) over seven days before and at the end of the intervention. Energy intake was calculated from food diaries over four days before and at the end of the intervention. Results are presented as mean ± SD. A paired Student's t-test assessed differences between baseline and after 12 weeks of intervention; * p<0.05, * p<0.01, *** p<0.001. An unpaired Student's t-test assessed differences between groups at baseline. *n* refers to the sample size in PD and PD+E for each measure. Data are presented as mean +/- SD.
E% = energy percentage.

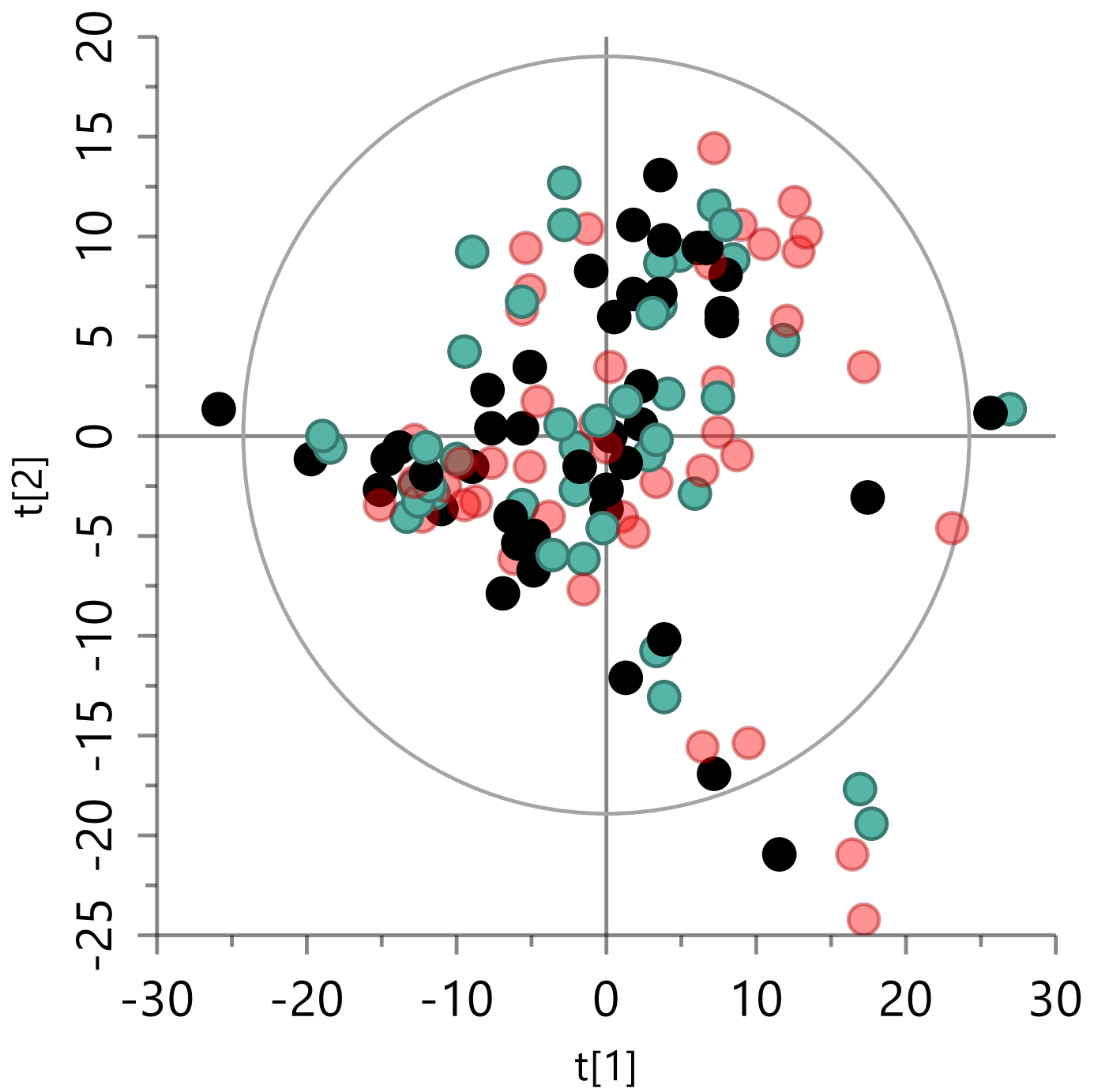

ESM Fig. 1 shows the principal component analysis (PCA) of the plasma lipidome comprising 225 lipids quantified by mass spectrometry in samples collected at baseline, immediately post-exercise, and during recovery. The first two principal components explained 21.4% (PC1, t[1]) and 13.0% (PC2, t[2]) of the total systematic variation in the dataset. Samples are coloured according to sampling time point: baseline (black), post-exercise (green), and recovery (red). No marked outliers or clustering patterns suggestive of technical variation related to instrumentation or sample handling were observed.

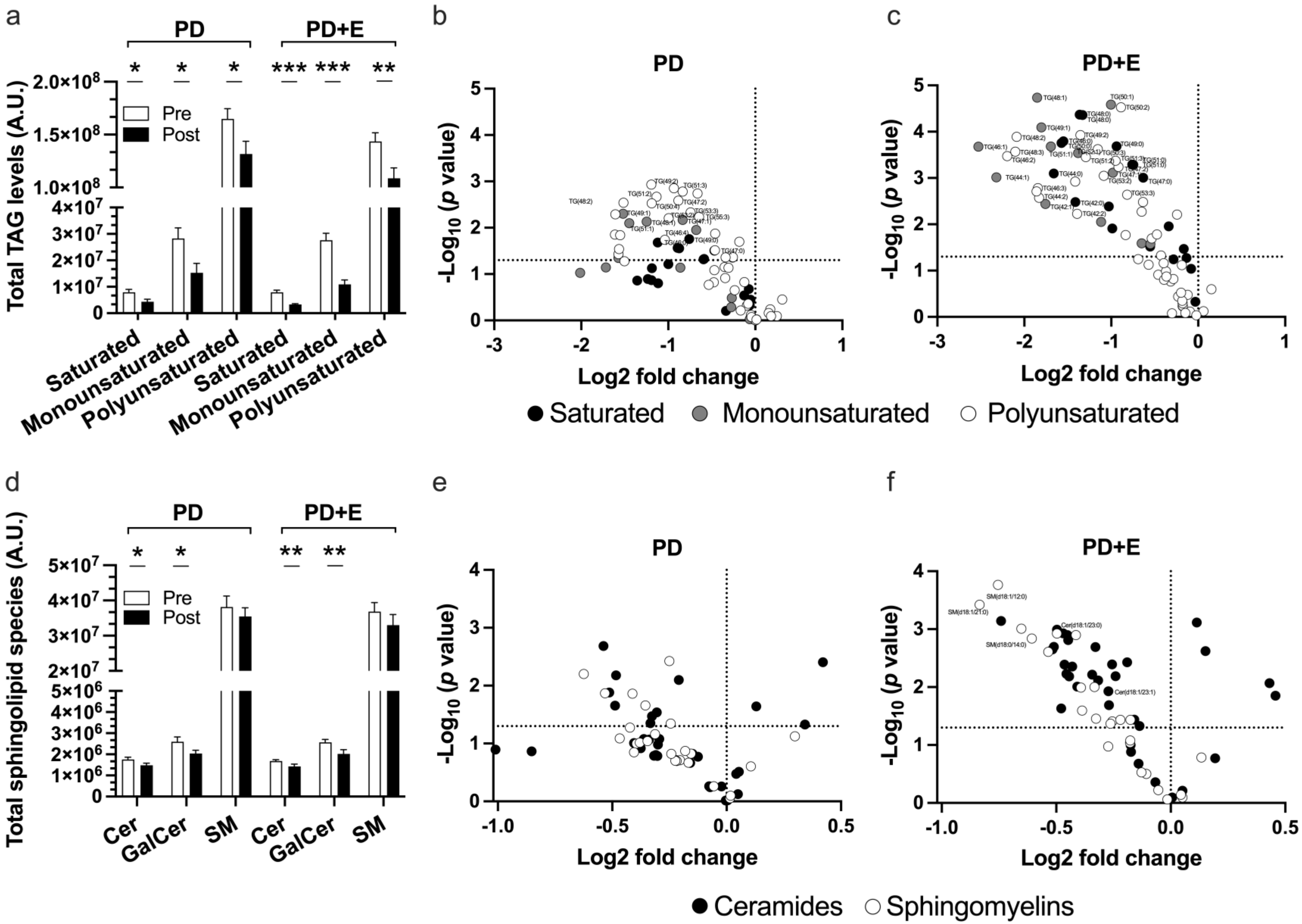
a
PD
PD+E
* * * *** *** **
Total TAG levels (A.U.)
Pre
Post
Saturated
Monounsaturated
Polyunsaturated
Saturated
Monounsaturated
Polyunsaturated
b
PD
-Log10 (p value)
Log2 fold change
c
PD+E
-Log10 (p value)
Log2 fold change
Saturated
Monounsaturated
Polyunsaturated
d
PD
PD+E
* * ** **
Total sphingolipid species (A.U.)
Pre
Post
Cer
GalCer
SM
Cer
GalCer
SM
e
PD
-Log10 (p value)
Log2 fold change
f
PD+E
-Log10 (p value)
Log2 fold change
Ceramides
Sphingomyelins

ESM Fig. 2 shows changes in resting plasma levels of triacylglycerols (TAG) and sphingolipids post- to pre-intervention. (a) Total plasma levels of saturated, monounsaturated, and polyunsaturated TAGs; (b and c) post- to pre-responses of individual TAGs; (d) total levels of ceramides (Cer), galactosylceramides (GalCer), and sphingomyelins (SM); (e and f) post- to pre-responses of individual sphingolipids. In volcano plots, the x-axis represents the magnitude and direction of change, while the y-axis indicates the significance of the change. Annotated lipids are those identified as different between time points according to the OPLS analysis and are significantly changed according to t-tests ($p<0.05$). The horizontal dotted line at 1.3 represents a significant change of $p = 0.05$. In A and D, a Student's t-test assessed within-group differences between baseline and after 12 weeks of intervention: * $p<0.05$, ** $p<0.01$, *** $p<0.001$. Results are presented as mean ± SEM. Cer, ceramide; GalCer, galactosylceramide; SM, sphingomyelin; TAG, triacylglycerols. In all panels: PD, palaeolithic-type diet ($n = 10$); PD+E, palaeolithic-type diet and exercise ($n = 11$). Data are presented as mean +/- SD.

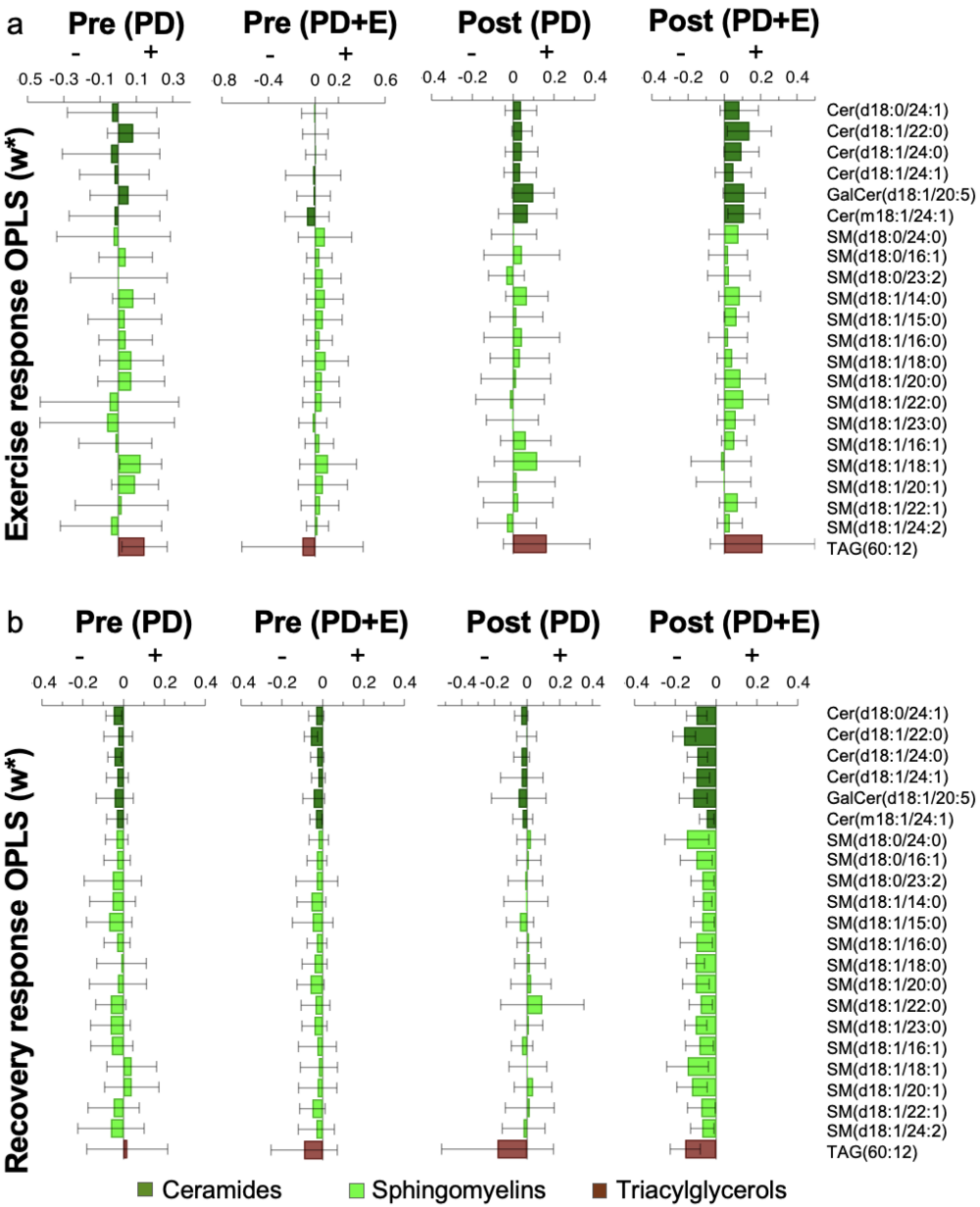
a
Pre (PD)
Pre (PD+E)
Post (PD)
Post (PD+E)
Exercise response OPLS (w*)
b
Recovery response OPLS (w*)
Cer(d18:0/24:1)
Cer(d18:1/22:0)
Cer(d18:1/24:0)
Cer(d18:1/24:1)
GalCer(d18:1/20:5)
Cer(m18:1/24:1)
SM(d18:0/24:0)
SM(d18:0/16:1)
SM(d18:0/23:2)
SM(d18:1/14:0)
SM(d18:1/15:0)
SM(d18:1/16:0)
SM(d18:1/18:0)
SM(d18:1/20:0)
SM(d18:1/22:0)
SM(d18:1/23:0)
SM(d18:1/16:1)
SM(d18:1/18:1)
SM(d18:1/20:1)
SM(d18:1/22:1)
SM(d18:1/24:2)
TAG(60:12)
Ceramides
Sphingomyelins
Triacylglycerols

ESM Fig. 3 shows changes in specific lipids that were altered by the intervention according to the OPLS model. (a) Plasma lipid responses to exercise in both groups pre- and post-intervention; (b) lipid responses to recovery. The main alterations that explain the variability of these lipids occur during recovery from exercise in the exercise group (PD+E). The x-axis shows the OPLS loading (w*), which describes the magnitude and direction of change for each lipid. Lipids that significantly change have a confidence interval that does not pass 0. OPLS, orthogonal partial least squares; Cer, ceramide; GalCer, galactosylceramide; SM, sphingomyelin; TAG, triacylglycerol. In all panels, PD, palaeolithic-type diet (*n* = 10); PD+E, palaeolithic-type diet and exercise (*n* = 11). Data are presented as mean +/- SD.
